# Inhibition of lipid droplet formation by Ser/Thr protein phosphatase PPM1D inhibitor, SL-176

**DOI:** 10.1371/journal.pone.0212682

**Published:** 2019-02-27

**Authors:** Rui Kamada, Nozomi Kimura, Fumihiko Yoshimura, Keiji Tanino, Kazuyasu Sakaguchi

**Affiliations:** 1 Laboratory of Biological Chemistry, Department of Chemistry, Faculty of Science, Hokkaido University, Sapporo, Japan; 2 School of Pharmaceutical Sciences, University of Shizuoka, Shizuoka, Japan; 3 Laboratory of Organic Chemistry II, Department of Chemistry, Faculty of Science, Hokkaido University, Sapporo, Japan; Kindai University, JAPAN

## Abstract

Obesity is a worldwide public health problem, which is associated with various severe diseases including diabetes, hypertension, atherosclerosis, and cancer. Recent studies have revealed that combination treatment of several different compounds using low doses is effective to reduce side effects. Thus, there is a need to develop an efficient inhibitor for reducing lipid droplets with a divergent target/pathway. Ser/Thr protein phosphatase PPM1D is involved in cellular metabolic processes and is a promising target for anti-obesity treatment. We have previously developed a potent and specific PPM1D inhibitor, SL-176. In this study, we demonstrated that significant reduction of lipid droplet formation in adipocytes by the PPM1D specific inhibitor, SL-176. Using Oil-red O staining and fluorescent imaging of lipid droplet, we found that treatment of SL-176 significantly suppressed lipid droplet formation of 3T3-L1 cells both in amount and in size. SL-176 also repressed mRNA and protein expression of PPARγ and C/EBPα, adipogenic markers, at nontoxic conditions. Thus, SL-176 is a unique and potent inhibitor of lipid droplet formation that acts via PPM1D, a novel target toward inhibiting adipocyte differentiation.

## Introduction

Obesity, a significant risk factor for multiple diseases including diabetes, heart disease, hypertension, and cancer, is becoming a global public health problem. Rates of obesity are rapidly increasing in all parts of the world. Thus, there are increasing demands for developing efficient and safe drugs toward obesity treatment. Obesity is induced by adipocyte hypertrophy and an increase in adipose tissue mass. Adipocytes, which have a central role in metabolism in the body, have large lipid droplets (LDs) in their cytosol to store energy including lipids and sugars[1]. LDs in adipocytes are a main storage of energy in the body and have an important role in regulating body metabolism[2]. LD formation in adipocytes is strictly mediated by a variety of proteins; particularly, peroxisome proliferator-activated receptor γ (PPARγ) and CCAAT/enhancer binding protein α (C/EBPα) are called master regulators of adipogenesis and lipid droplet formation in adipocytes[3, 4]. PPARγ has two different isoforms: PPARγ1 and PPARγ2, which only differ in the N-terminal 30 amino acids. PPARγ1 is expressed in many types of tissues, whereas PPARγ2 is expressed in adipose tissue.

Previous studies have shown that various natural compounds decreased lipid droplets in adipocytes[5, 6]. Several compounds such as epigallocatechin gallate (EGCG) and baicalein decreased lipid accumulation; EGCG activates AMP-activated protein kinase (AMPK) and baicalein enhances COX-2 expression, leading to decreased PPARγ or C/EBPα expression. However, most compounds have unknown mechanisms and unknown target pathways of inhibitory activity for lipid droplet formation. Recent studies have shown that combination therapy with compounds that target different adipocyte pathways is required for efficient treatment[7, 8]. Thus, it is necessary to develop potent and novel inhibitory compounds which acts via different targets/pathways.

PPM1D (Protein phosphatase magnesium-dependent 1δ, also known as Wip1, PP2Cδ), a p53-inducible Ser/Thr phosphatase, is involved in cellular homeostasis via negative feedback of the p53-pathway[9]. PPM1D also has multiple functions in cellular differentiation and immune responses[10, 11]. Recently, it has been reported that PPM1D has a critical role in metabolism. *PPM1D*^*-/-*^ *-apoE*^*-/-*^ mice showed anti-atherosclerosis and anti-obesity compared with WT mice[12]. Moreover, *PPM1D*^*-/-*^ mice exhibited insulin resistance and suppression of weight gain[13]. It also was reported that PPM1D is involved in adipogenesis and fat accumulation via direct dephosphorylation of PPARγ[14]. Recently, a specific PPM1D inhibitor, SL-176, has been developed in our group[15]. SL-176 potently and specifically inhibits the phosphatase activity of PPM1D. Moreover, SL-176 greatly suppressed proliferation of PPM1D-overexpressing cancer cells while it had no effect on cells which express normal level of PPM1D. Here we report a novel and potent inhibition activity of adipocyte differentiation by SL-176, which suppresses PPM1D phosphatase activity and leads to down-regulation of the PPARγ pathway.

## Results and discussion

**Fig 1:**
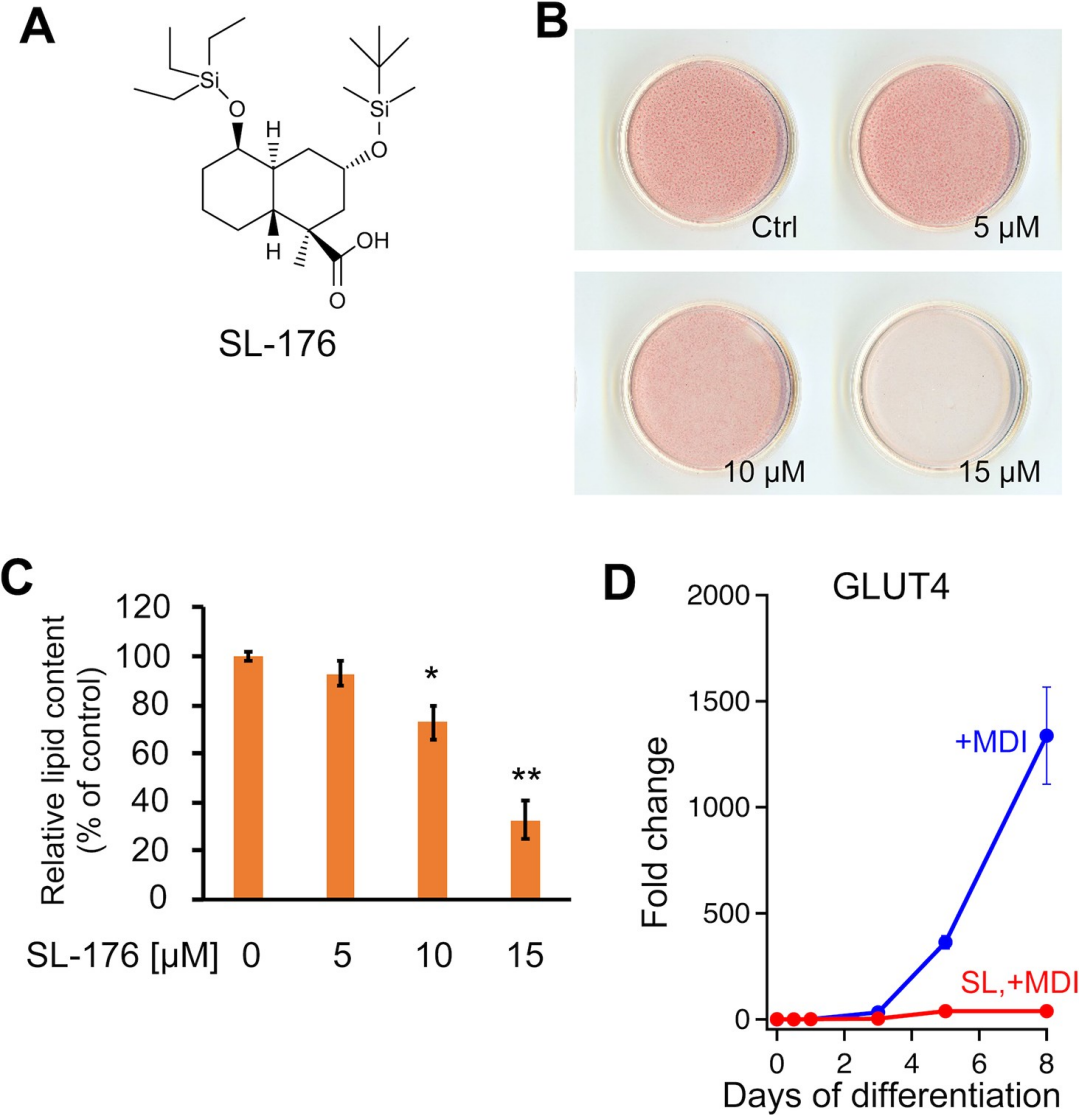
PPM1D inhibitor SL-176 suppresses cellular lipid droplet accumulation and adipocyte differentiation. (A) PPM1D inhibitor SL-176. (B, C) Quantification of lipid droplets in 3T3-L1 cells treated with the indicated concentrations of SL-176. Differentiated 3T3-L1 cells on Day 8 were stained with Oil Red O. (C) Absorbance of Oil Red O extract was measured at 490 nm. Data are mean ± S.D. values and obtained by three independent samples in each conditions (*P<0.05 **P<0.01 respectively, paired Student's t-test) (D) mRNA expression of the adipocyte marker by RT-qPCR. Cells were treated with differentiation medium (MDI) with or without 10 μM of SL-176. Blue, with MDI; red, 10 μM of SL-176 with MDI. The data were normalized by actin and expressed as fold change. Values are the mean ± range of duplicates. Representative data from one of at least three independent experiments are shown.

**Fig 2:**
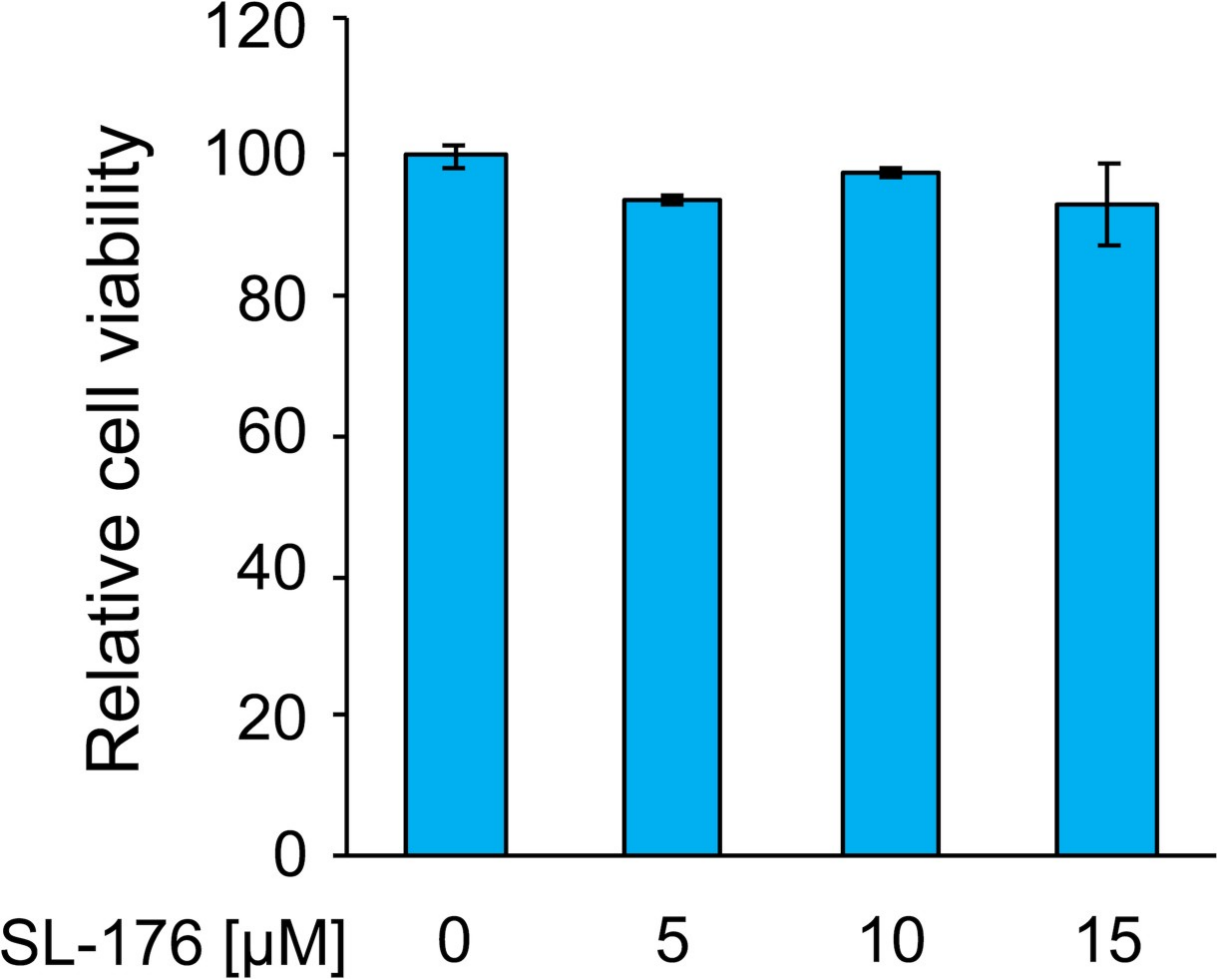
Cell viability of 3T3-L1 preadipocytes after treatment with the indicated concentrations of SL-176 for 24 h was measured by MTS assay. The data represent the mean ± S.D. of triplicate samples.

To analyze whether the PPM1D inhibitor SL-176 suppresses lipid droplet formation in adipocytes, 3T3-L1 preadipocytes were induced to differentiate into adipocyte in the absence or presence of SL-176. After 8-days induction of adipocyte-differentiation, 3T3-L1 cells were stained by Oil Red O for quantifying of amounts of lipid droplets. 3T3-L1 adipocyte cells increased lipid droplets in cells. SL-176 dramatically decreased lipid droplets in differentiated 3T3-L1 adipocyte cells on Day8 in a dose-dependent manner (Fig 1). Quantitative analysis showed that treatment with 15 μM of SL-176 significantly decreased the amount of lipid droplets to 32% compared with control cells (Fig 1C). On the other hand, treatment with SL-104[16] and SL-188 in which silyl groups, essential units for inhibitory activity against PPM1D, were removed, did not affect the amount of lipid droplets in 3T3-L1 adipocyte cells (S1 Fig). Furthermore, SL-176 dramatically decreased the expression of adipocyte marker, GLUT4 compared with the condition in the absence of SL-176 (Fig 1D). It is worth noting that SL-176 did not affect cell viability of 3T3-L1 cells confirmed by the MTS assay after treatment with SL-176 for 24 h (Fig 2). These results indicated that the decrease of lipid droplet formation by SL-176 was not due to induce cell death. These results revealed that SL-176 has a novel biological activity, which suppresses lipid droplet formation and adipocyte differentiation.

**Fig 3:**
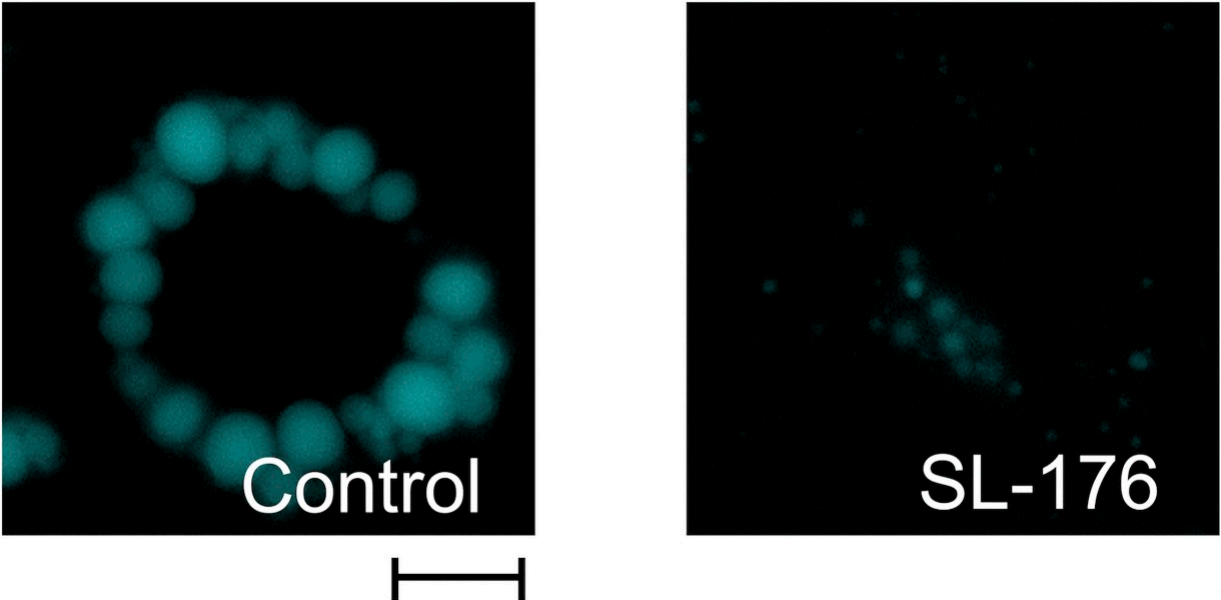
SL-176 significantly reduced the size of lipid droplets in 3T3-L1 cells. After treatment of SL-176 during adipocyte differentiation (from Day0 to Day8), lipid droplets in differentiated 3T3-L1 cells on Day 8 were stained by Monodansylpentane. Scale bar = 10 μm.

Fluorescence imaging of lipid droplets revealed that SL-176 treatment drastically decreased lipid droplet sizes (Fig 3 and Table 1). We chose to examine lipid droplets after 8 days of differentiation as this time is typical for these types of experiments. The average size of lipid droplets clearly decreased from 2.95 μm in control cells to 1.71 μm in SL-176 treated cells. The percentage of lipid droplets with diameters > 4 μm in SL-176-treated cells was significantly decreased to only 1.6%, whereas the percentage in control cells was 21.3%. Moreover, the fraction of lipid droplets smaller than 2 μm was 36% in control cells, and it became 73.6% after SL-176 treatment. SL-104 did not affect lipid droplet sizes in 3T3-L1 cells (S2 Fig). This indicates that PPM1D inhibition significantly decreased lipid droplet size. Large lipid droplets were more slowly degraded than small lipid droplets[17]. Moreover, enlargement of lipid droplet causes hypertrophy of adipocyte and obesity. Therefore, it is worthy to note that SL-176 reduced the size of lipid droplet in adipocyte cells.

**Table 1:** LD size distribution in SL-176 treated cells.

	Average size*	Size distribution of LDs (%)				
	[μm]	0–2 μm	2–4 μm	4–6 μm	6–8 μm	>8 μm
Control	2.95 ± 0.17	36.0	42.8	15.1	4.1	2.1
SL-176	1.71 ± 0.14	73.6	24.8	1.6	0.0	0.0

LD size distribution in 3T3-L1 cells. LD diameters were calculated by Image J.

*The average size of lipid droplet represents the mean ± S.D.

**Fig 4:**
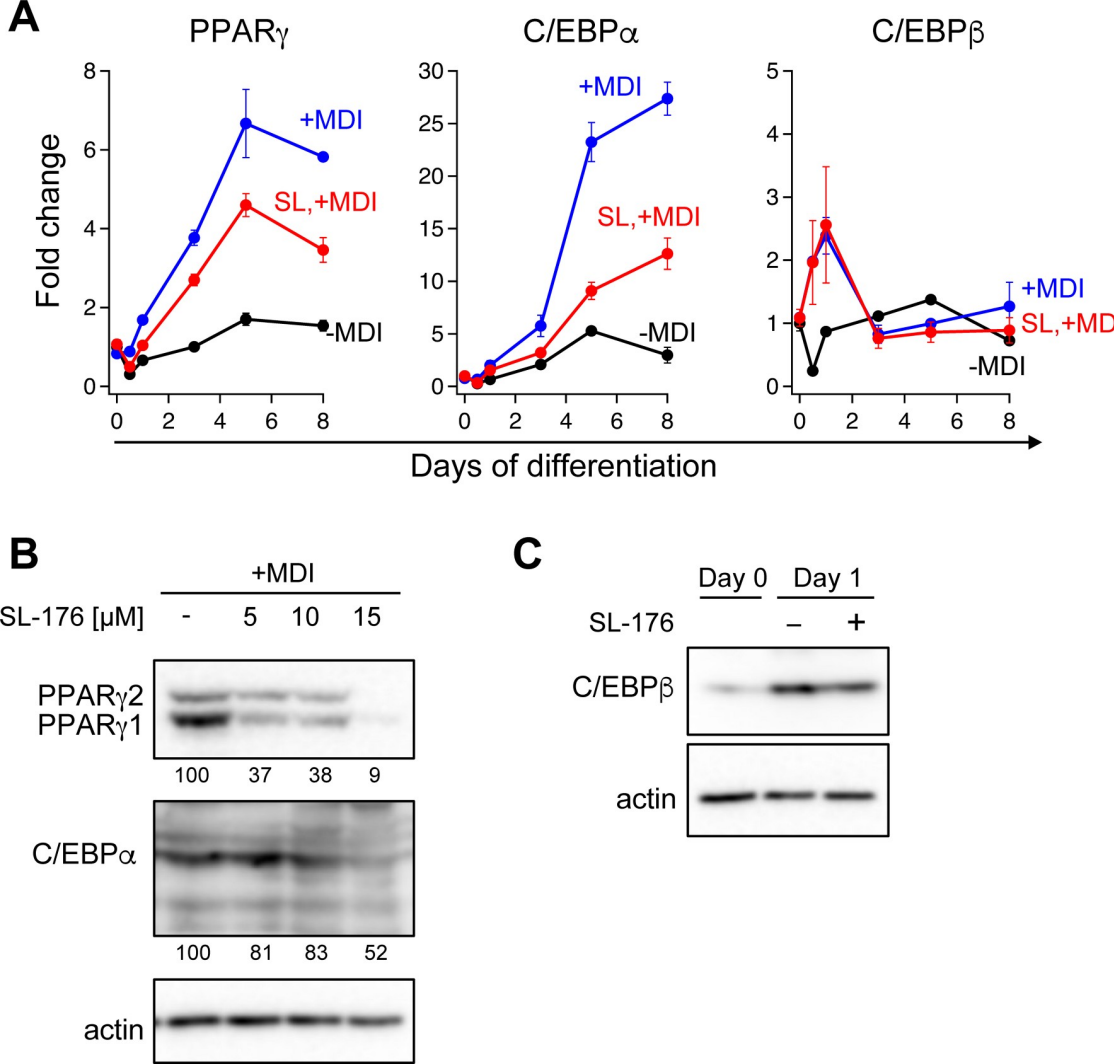
Expression of adipocyte markers were reduced by SL-176. (A) mRNA expression of the indicated genes was measured by RT-qPCR. Cells were treated with differentiation medium (MDI) with or without 10 μM of SL-176. Black line, without MDI (control); blue line, with MDI; red line, 10 μM of SL-176 with MDI. The data were normalized by actin and expressed as fold change. Values are the mean ± range of duplicates. Representative data from one of at least three independent experiments are shown. (B) Protein expression levels of PPARγ1/2 and C/EBPα in 3T3-L1 cells on Day 8 in the presence of 0, 5, 10, 15 μM SL-176 were analyzed by Western blotting. The intensity of the protein was shown below the western panel as fold change compared with intensity of 0 μM of SL-176. The values were normalized by of actin values. (C) C/EBPβ protein expression in 3T3-L1 cells on Day1-differentiated in the presence of 10 μM SL-176.

During adipocyte differentiation, PPARγ, C/EBPα, and C/EBPβ, adipogenic transcription factors, were induced to increase their expression levels. Quantitative analysis of mRNAs during differentiation of 3T3-L1 cells in the presence of SL-176 indicated that PPARγ and C/EBPα were remarkably reduced by half or less than half in compared with control (Fig 4A). Protein expression levels of both PPARγ isoforms and C/EBPα also were greatly decreased in 3T3-L1 cells by SL-176 treatment (Fig 4B). On the other hand, SL-176 hardly affected C/EBPβ expression both at the mRNA or protein level (Fig 4A–4C). In adipocyte differentiation, C/EBPβ and C/EBPδ are induced during the first stages of the differentiation. C/EBPβ and C/EBPδ directly induce transcription of the PPARγ and C/EBPα genes, the master regulators of adipogenesis. SL-176 suppressed PPARγ and C/EBPα expression, possibly by inhibiting transcriptional activity of C/EBPβ and/or C/EBPδ. Because PPARγ and C/EBPα is essential for adipogenesis, SL-176 suppressed adipocyte differentiation probably by inhibiting induction of PPARγ and C/EBPα.

Here, we demonstrated that the PPM1D-specific inhibitor SL-176 has a strong inhibitory activity against lipid droplet formation in adipocytes with a higher degree of inhibition compared with various biomaterials that inhibit adipocyte differentiation[5]. We also showed that PPM1D inhibition significantly suppressed induction of PPARγ and C/EBPα expression in response to adipocyte differentiation, leading to a decrease in lipid droplet formation. In previous work, naturally-occurring compounds which showed inhibitory activity against adipocyte differentiation, have the ability to induce apoptosis in maturing pre-adipocytes though regulating ERK1/2 phosphorylation, activation of the mitochondrial pathway, AMPK activation or anti-oxidant activity[5]. However, for most of these compounds, target proteins were still unclear. We have demonstrated that PPM1D is a novel target for suppressing lipid droplet formation. PPM1D inhibitor SL-176 significantly reduced lipid droplet formation probably via inhibiting the PPM1D-C/EBPα-PPARγ differentiation pathway without any toxicity. It is reported that PPM1D knockout mice exhibit insulin resistance[13]. Another possible mechanism is that SL-176 suppress adipocyte differentiation by affecting insulin sensitivity. Additionally, SL-176 has a completely different and unique molecular architecture as an anti-obesity compound. This provides insights into PPM1D inhibitor SL-176 as a lead compound for novel and safe anti-obesity drugs, although it may require precaution of affecting insulin response.

Recently, multiple anti-obesity drugs has been approved by the US Food and Drug Administration (FDA)[7]. Some of these drugs have side effects such as nausea, headache, and dry mouth. Combination treatment by using lower doses of two or more anti-obesity drugs that work in different pathway/targets promise to reduce such side-effects and achieve efficient reduction of adipocytes. For efficient combination treatment for anti-obesity, it is extremely important to develop potent anti-obesity compounds, which exert their activity via different targets or pathways from conventional anti-obesity drug. Thus, our novel and potent inhibitor targeting PPM1D phosphatase activity for lipid droplet formation will be an effective compound for anti-obesity therapy. PPM1D is known as a promising target for cancer therapy. PPM1D is overexpressed in various tumors including breast cancer and ovarian cancer[18]. Recent study has revealed that lipid droplets are a possible potential target for cancer treatment. The number of lipid droplet increases in tumor cells[19]. Thus, PPM1D inhibitor SL-176 also is a lead compound for development of an anti-cancer drug, which affects via dual pathways.

## Materials and methods

### Compounds

We confirmed that SL-176 strongly inhibited mouse PPM1D phosphatase activity at the maximal inhibitory concentration (IC_50_) of 112 ± 12 nM, the same level as the IC_50_ for human PPM1D (S3 Fig). Phosphatase activity was assayed by measuring the released free phosphate by BIOMOL GREEN (Enzo life sciences) as previously described. Antibodies used: anti-C/EBPβ H-7, anti-PPARγ E-8 and anti-C/EBPα D-5 antibodies, Santa Cruz Biotechnology; anti-actin C-4 antibody, Ab-1, and anti-mouse IgG-HRP, GE-healthcare; anti-rabbit IgG-HRP, Cell Signaling.

### Cells

3T3-L1 mouse fibroblasts were obtained from the Japanese Collection of Research Bioresource and grown in Dulbecco’s modified Eagle’s medium supplemented with 10% new bovine calf serum at 37°C in an atmosphere of 5% CO_2_. 3T3-L1 cells were seeded in the density of 8 x 10^4^ cells per 35 mm well. Two days after 100% confluence cells (Day0), cells were cultured in Differentiation Medium (MDI) [DMEM containing 10% v/v fetal bovine serum (FBS) with 100 units/mL Penicillin, 100 μg/mL Streptomycin (P/S) (Thermo Fisher Scientific, CA, USA). with 1.0 μg/mL Insulin, 1.0 μM Dexamethasone, 0.5 mM 3-isobutyl-1-methylxanthine (IBMX)]. Cells were placed in Adipocyte Maintenance Medium [DMEM containing 10% FBS with P/S with 1.0 μg/mL Insulin] on Day1. Media were exchanged to Adipocyte Maintenance Medium every two days until Day8 of induction of differentiation.

### Oil Red O staining

3T3-L1 cells were cultured at the density of 8 x 10^4^ cells per 35 mm well. Oil Red O solution was 0.3% Oil Red O/2-propanol: H_2_O = 6:4 and was filtered through a 0.45 μm PVDF filter (Millipore). 5% v/v 60% Isopropanol/H_2_O was added to this filtered solution. After Day8 of differentiation, cells were washed with PBS one time, 60% 2-propanol one time, and then added to an Oil Red O solution for 30 minutes. After staining, cells were washed with 60% 2-propanol/H_2_O three times. After drying dishes overnight, extraction of Oil Red O was performed with 100% 2-propanol for 60 minutes once, and for 10 minutes twice. For quantification, absorbance of Oil Red O extract was measured at 490 nm with a microplate reader. Cells were harvested with lysis buffer (50 mM Tris-HCl (pH6.8), 2% SDS, 10% glycerol, 100 mM dithiothreitol), and lysate cells were centrifugation at 15000 rpm for10 minutes.

### Cytotoxicity assay

MTS assays were performed to evaluate the effect of SL-176 on cell viability of 3T3-L1 cells following the manufacture’s protocol. Briefly, 3T3-L1 cells were seeded at a density of 4 x 10^3^ cells per 96-well plate. After 24 h from seeding, cells were incubated with 0, 5, 10, 15, 20 μM SL-176 for 24 h. After incubation with Cell Titer 96@Aquous One Solution Cell Proliferation Assay for 30 min, the absorbance at 490 nm was measured with a microplate reader.

### Lipid droplets imaging

3T3-L1 cells were seeded in the density of 1.6 x 10^4^ cells on micro cover glasses in a 24-well plate and cultured as above. On Day8 of differentiation, cells were fixed with 3.5% formaldehyde for 1 h, washed in PBS, and permeabilized with 0.2% Triton X-100/PBS for 15 min. After that, cells were incubated with 10 μM Monodansylpentane (Abgend) for 1 h, and then observed by fluorescence microscopy (BZ-9000, KEYENCE, Osaka, Japan).

### Quantification of lipid droplet size

The diameter of Lipid droplets was measured in nine images of MDH-stained cells (approximately total of 100~200 cells). Lipid droplets in images were circled and painted in black by using PowerPoint. Figs which overlap each other were removed. Obtained images were used as binarized images. The size of circles was measured by ImageJ.

### Western blotting

3T3-L1 cells were seeded in the density of 8 x 10^4^ cells per 35 mm μ-dish and cultured as above. Cells on each day of differentiation were lysed with buffer (50 mM Tris-HCl (pH6.8), 2% SDS, 10% glycerol, 6% β-mercaptoethanol), and heated at 100°C for 5 minutes. Cell lysates were separated with SDS-PAGE and transferred to polyvinylidene difluoride membranes (Millipore). Transferred membranes were blocked with 5% or 2% milk/TBST. Visualization of proteins were by enhanced chemiluminescence with the above antibodies.

### Quantitative RT-PCR

3T3-L1 cells were seeded in the density of 1.6 x 10^4^ cells per 24-well plate. Induction of 3T3-L1 differentiation was performed as above. Cells were washed with PBS and lysed with TRIzol reagent (Life technologies). Total RNA was purified according to the manufacturer’s protocol. Total RNA (0.5 μg) was then reverse transcribed using the PrimerScript II 1st strand cDNA Synthesis Kit (TaKaRa, Kyoto, Japan) with random hexamer primers according to the manufacturer’s protocols. Quantitative PCR was performed with a CFX96 Touch realtime PCR detection system (Bio-Rad, CA, USA) using iTaq Universal SYBR Green Supermix (Bio-Rad, CA, USA). Primers used for quantitative PCR are listed in S1 Table.

## Supporting information

S1 Fig Quantification of lipid droplets with 10 μM SL-104 and SL-188. Amount of lipid droplets was quantified by Oil Red O staining.
(TIF)

S2 Fig Imaging of lipid droplets by Monodansylpentane. 3T3-L1 cells were differentiated for 8 days with 15 μM SL-104.
(TIF)

S3 Fig Inhibitory activity of SL-176 for His-mousePPM1D (1–413) *in vitro*. Phosphatase activity of His-mousePPM1D (1–413) for p53-derived peptide was measured.
(TIF)

S1 Table Primer list. (PDF)

## Abbreviations

AMPK: AMP-activated protein kinase
PPARγ: peroxisome proliferator-activated receptor γ
C/EBP: CCAAT/enhancer binding protein
EGCG: epigallocatechin gallate
LD: Lipid droplet
IBMX: 3-isobutyl-1-methylxanthine
