# Supplementary figures and images for: Inhibition of lipid droplet formation by Ser/Thr protein phosphatase PPM1D inhibitor, SL-176
Source: PLoS One. 2019 Feb 27;14(2):e0212682. doi: 10.1371/journal.pone.0212682 (PMC6392468; doi:10.1371/journal.pone.0212682)

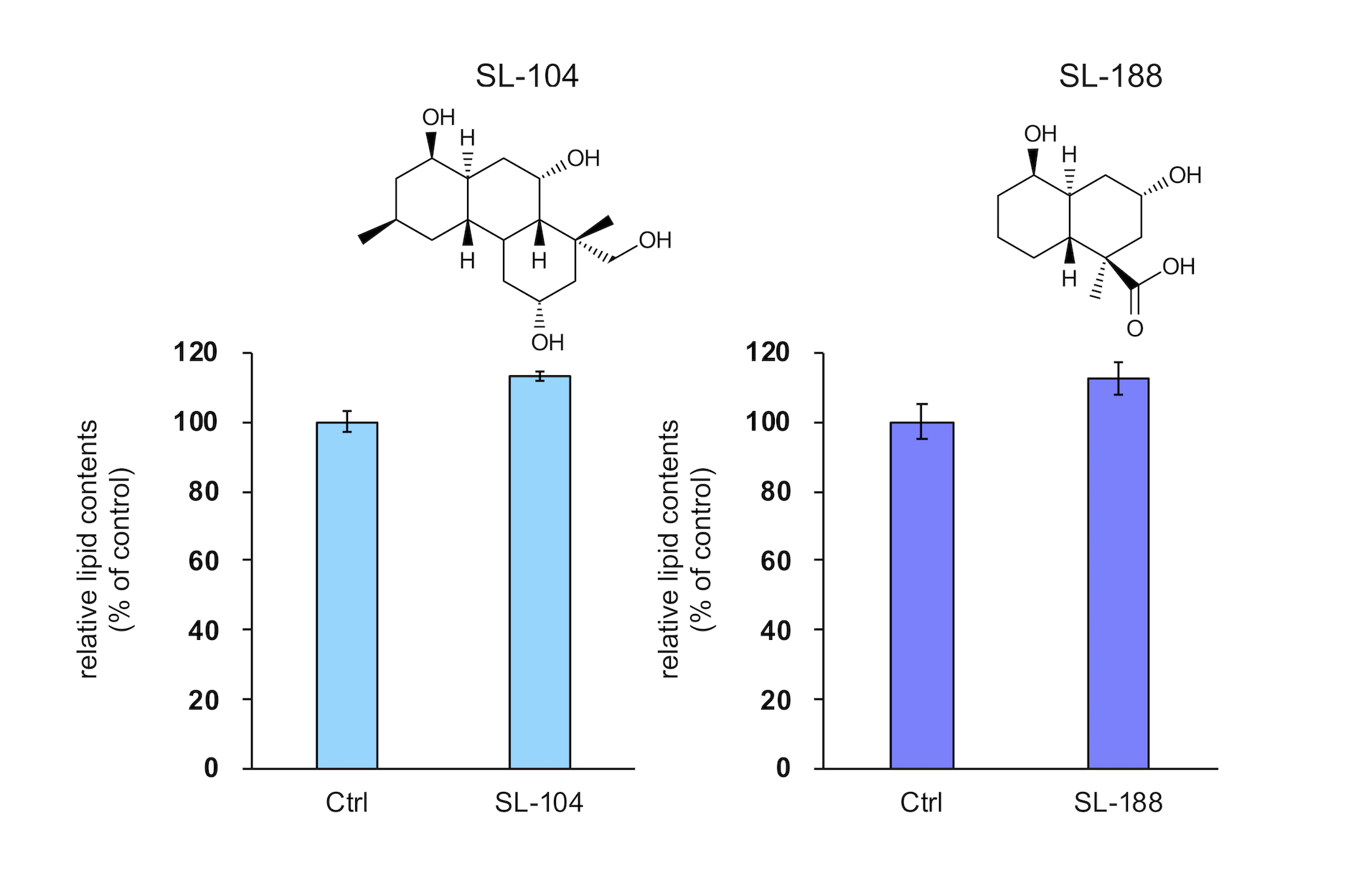

Supplement: S1 Fig — Amount of lipid droplets was quantified by Oil Red O staining. (TIF) [file pone.0212682.s001.tif]

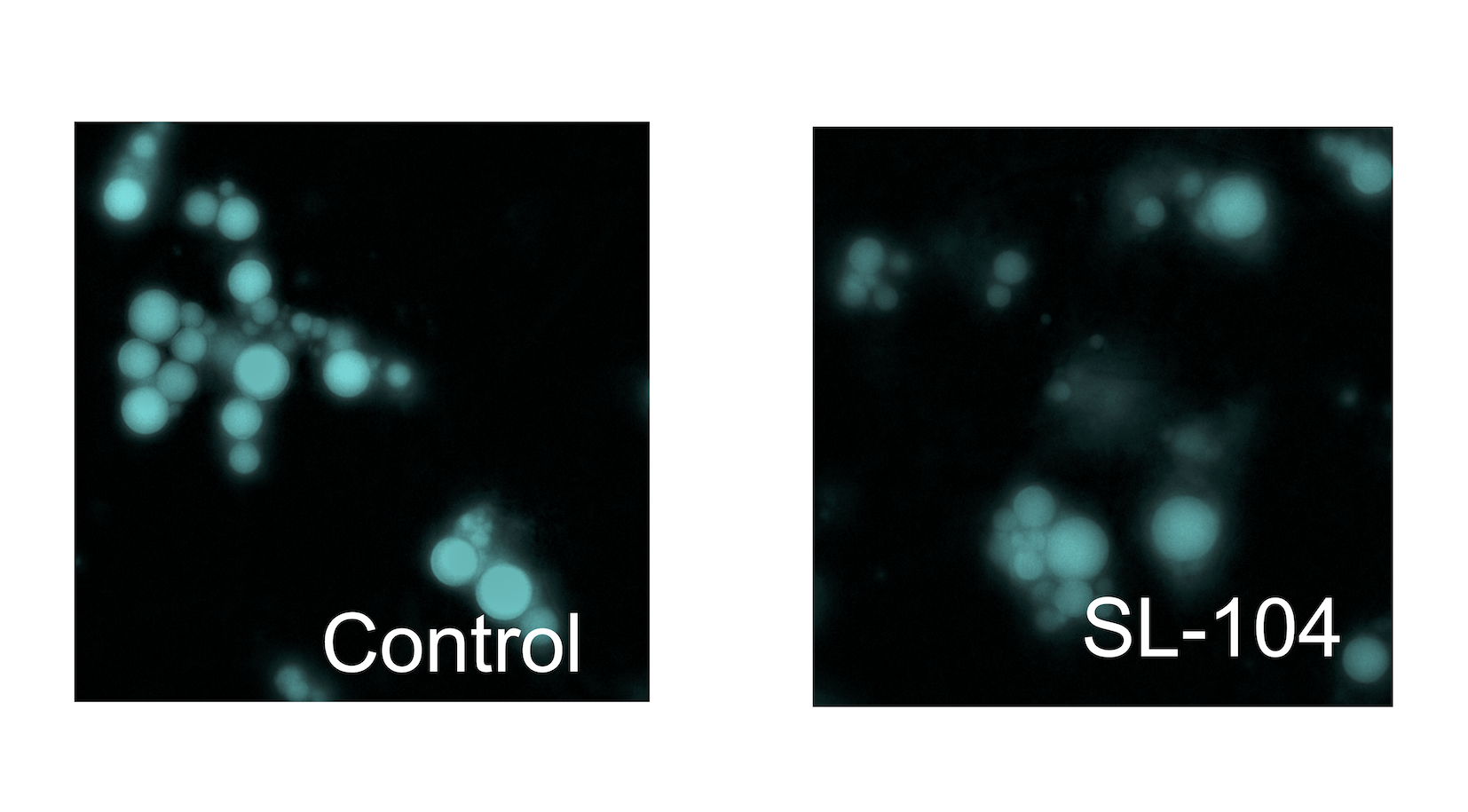

Supplement: S2 Fig — 3T3-L1 cells were differentiated for 8 days with 15 μM SL-104. (TIF) [file pone.0212682.s002.tif]

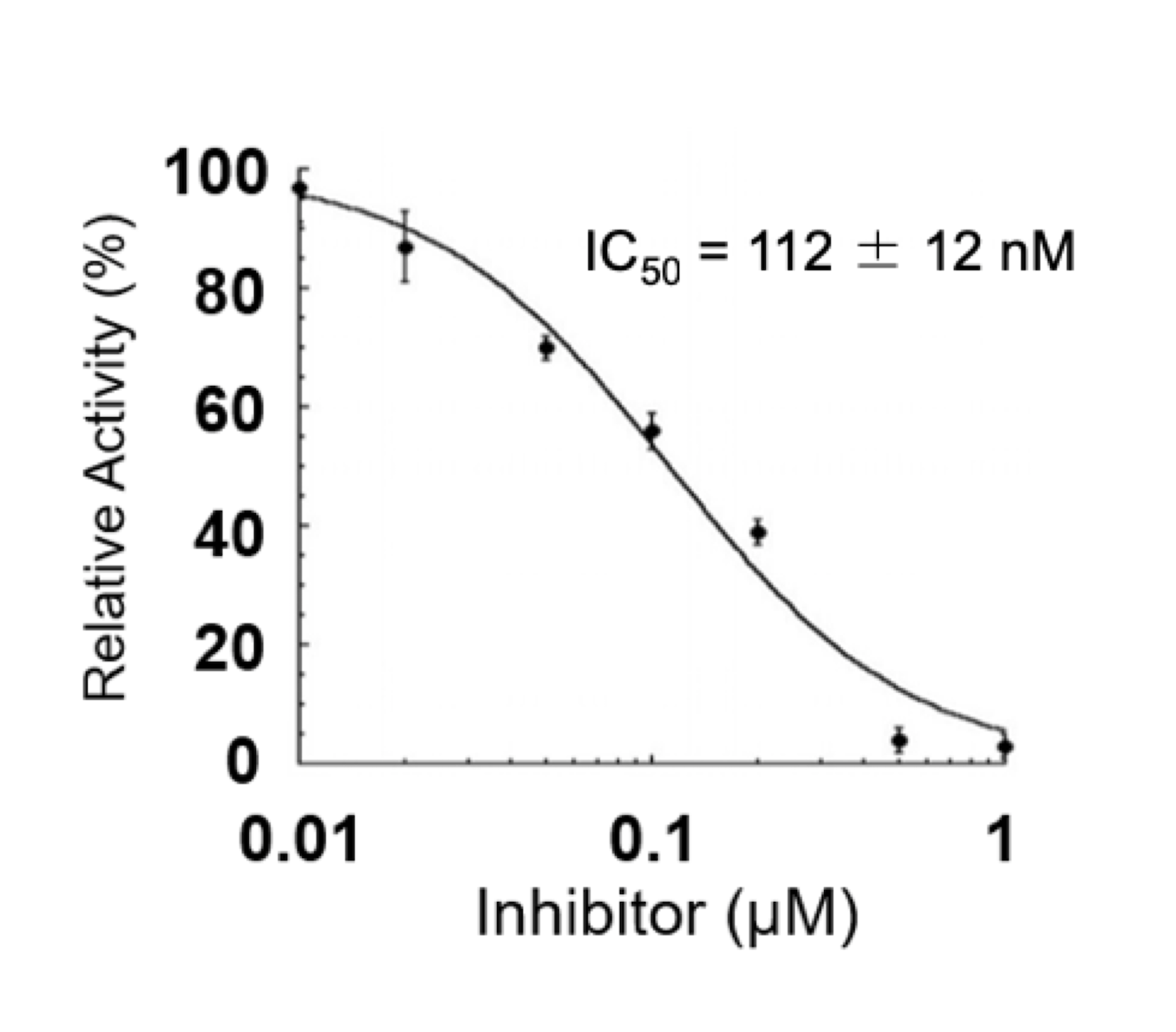

Supplement: S3 Fig — Phosphatase activity of His-mousePPM1D (1–413) for p53-derived peptide was measured. (TIF) [file pone.0212682.s003.tif]
