# Supplementary material for: Inhibition of lipid droplet formation by Ser/Thr protein phosphatase PPM1D inhibitor, SL-176
Source: PLoS One. 2019 Feb 27;14(2):e0212682. doi: 10.1371/journal.pone.0212682 (PMC6392468; doi:10.1371/journal.pone.0212682)
Supplement: S1 Table — (PDF) [file pone.0212682.s004.pdf]

Table S1 Primer list

Gene	Sequence
β-actin	Forward: GGCTGTATTCCCCTCCATCG
	Reverse: CCAGTTGGTAACAATGCCATGT
C/EBPα	Forward: CAAGAACAGCAACGAGTACCG
	Reverse: GTCACTGGTCAACTCCAGCAC
C/EBPβ	Forward: TGATGCAATCCGGATCAA
	Reverse: CACGTGTGTTGCGTCAGT
PPARγ	Forward: CCATTCTGGCCCACCAAC
	Reverse: AATGCGAGTGGTCTTCCATCA
GLUT4	Forward: TCGTCATTGGCATTCTGGTTG
	Reverse: AGCTCGTTCTACTAAGAGCACG
